# High Hydrostatic Pressure Therapy Annihilates Squamous Cell Carcinoma in a Murine Model

**DOI:** 10.1155/2020/3074742

**Published:** 2020-03-07

**Authors:** Toshihito Mitsui, Naoki Morimoto, Atsushi Mahara, Sharon Claudia Notodihardjo, Tien Minh Le, Maria Chiara Munisso, Natsuko Kakudo, Tetsuji Yamaoka, Kenji Kusumoto

**Affiliations:** ^1^Department of Plastic and Reconstructive Surgery, Kansai Medical University, 2-5-1 Shin-Machi, Hirakata City, Osaka 573-1191, Japan; ^2^Department of Plastic and Reconstructive Surgery, Graduate School of Medicine, Kyoto University, Sakyou-Ku Yoshida Konoe-Chou, Kyoto City, Kyoto 606-8501, Japan; ^3^Department of Biomedical Engineering, National Cerebral and Cardiovascular Center Research Institute, 6-1 Kishibe Shin-Machi, Suita City, Osaka 564-8565, Japan

## Abstract

Cutaneous squamous cell carcinoma (cSCC) is one of the most common skin cancers. In the treatment of cSCC, it is necessary to remove it completely, and reconstructive surgery, such as a skin graft or a local or free flap, will be required, depending on the size, with donor-site morbidity posing a burden to the patient. The high hydrostatic pressure (HHP) technique has been developed as a physical method of decellularizing various tissues. We previously reported that HHP at 200 MPa for 10 min could inactivate all cells in the giant congenital melanocytic nevus, and we have already started a clinical trial using this technique. In the present study, we explored the critical pressurization condition for annihilating cSCC cells *in vitro* and confirmed that this condition could also annihilate cSCC *in vivo*. We prepared 5 pressurization conditions in this study (150, 160, 170, 180, and 190 MPa for 10 min) and confirmed that cSCC cells were killed by pressurization at ≥160 MPa for 10 min. In the *in vivo* study, the cSCC cells inactivated by HHP at 200 MPa for 10 min were unable to proliferate after injection into the intradermal space of mice, and transplanted cSCC tissues that had been inactivated by HHP showed a decreased weight at 5 weeks after implantation. These results suggested that HHP at 200 MPa for 10 min was able to annihilate SCC, so HHP technology may be a novel treatment of skin cancer.

## 1. Introduction

Cutaneous squamous cell carcinoma (cSCC) is one of the most common skin cancers, accounting for 20% of all nonmelanoma skin cancers and posing a deadly threat, due to its ability to metastasize to any organ in the body [1]. Various treatments for cSCC have been developed, such as surgical excision, radiation therapy, chemotherapy, epidermal growth factor receptor inhibitors, and immune checkpoint blockers [2]. In the treatment of the cSCC tumor, it is important to remove cSCC cells completely [3]. Reconstructive surgery, such as a skin graft or a local or free flap, will be required, depending on the defect left after resection [4, 5]. When we reconstruct skin defects after removal surgery, donor-site morbidity is a major issue for patients. A less invasive procedure is therefore desirable in the treatment of cSCC.

The high hydrostatic pressure (HHP) technique was developed as a physical method of decellularizing various tissues, such as the cornea and uterine tissue [6, 7]. We previously reported that HHP at 200 MPa for 10 min was able to inactivate all cells in the giant congenital melanocytic nevus (GCMN), and we have already started a clinical trial using this technique. In our clinical trial for GCMN, the nevus inactivated by HHP was used as an autologous dermis to cover full-thickness skin defects after its removal instead of autologous skin grafting [8]. Similar with GCMN, cSCC is also a type of skin tumor, so we expected that the skin defect after the removal of cSCC can be repaired using the inactivated cSCC tissue itself via the HHP technique.

Regarding the pressure and pressurization time needed to induce cell death, HHP between 150 and 200 MPa for 10 min can inactivate cells completely, as determined in our previous *in vitro* and *in vivo* experiments using a porcine autograft model [9–11]. In the present study, we first evaluated the pressurization conditions necessary to kill cSCC cells *in vitro* and then prepared a cSCC model using NOD.Cg-Prkdc^acid^Il2rg^tm1Wjl^/SzJ (NSG) mice and confirmed whether cSCC could be treated using this pressurization condition.

## 2. Materials and Methods

### 2.1. Ethics Statements

All animal experiments in this study were conducted at Kansai Medical University in accordance with the Guidelines for Animal Experiments established by the Ministry of Health, Labor and Welfare of Japan and by Kansai Medical University, Japan. The number of animals used in this study was kept to a minimum, and the protocol was approved by the Animal Research Committee of Kansai Medical University (Approval Number: 18-049).

### 2.2. Animals

NOD.Cg-Prkdc^acid^Il2rg^tm1Wjl^/SzJ (NSG) mice were purchased from Charles River Laboratories International, Inc. (Takatsuki, Japan). All mice were used at eight weeks of age according to the standard practices under an approved protocol. NSG mice were fed and housed in individual cages inside the institutional animal facility.

### 2.3. Cell Line and Culture Condition

We purchased and prepared cutaneous cell carcinoma A431 cells (cSCC; Cat. No. EC85090402; DS Pharma Biomedical Co., Ltd., Osaka, Japan). cSCC cells were cultured using Dulbecco's modified Eagle's medium (DMEM; “Nissui” 1; Nissui Pharmaceutical Co., Ltd., Tokyo, Japan) containing 10% fetal bovine serum (FBS; HyClone, Logan, UT, USA) and 1% antibiotic/penicillin and streptomycin solution (MP Biomedicals, LLC, Solon, OH, USA) at 37°C, 95% humidity, and 5% carbon dioxide. The medium of cSCC cells was changed every 3 or 4 days until confluency, at which point cells were washed with phosphate-buffered saline (-) (PBS(-); Takara Bio Inc., Kusatsu, Japan) and then dissociated using TrypLE™ Express (Life Technologies Co., Ltd.) and passaged. After 3 to 8 passages, 1 × 10^6^ cells were suspended in 1 mL of CELLBANKER® 1 plus (Nippon Zenyaku Kogyo Co., Ltd., Fukushima, Japan) and cryopreserved until subsequent experiments. We evaluated the pressurization condition that killed cells completely using the live/dead assay, morphological observation of the cultured cells, the water-soluble tetrazolium salt (WST) assay, and animal experiments.

### 2.4. Live/Dead Staining of Cells without Pressurization or after Pressurization

We prepared the live/dead staining working solution (Live/Dead Reduced Biohazard Viability/Cytotoxicity Kit #1; Life Technologies Co., Ltd.) by mixing Component A (SYTO10) and Component B (ethidium homodimer II (EthD-II)) according to the manufacturer's instructions.

Cryopreserved cSCC cells were rapidly thawed in a water bath, and 1 × 10^6^ cells each were seeded and cultured on a 10- or 15 cm culture dish with the respective culture medium. After reaching subconfluence, the cells were dissociated using TrypLE™ Express, and cell suspensions of 1 × 10^6^ cells/mL in the respective culture medium were prepared. The cell suspension was then packed in a plastic bag, and six bags were prepared for each kind of cell. One bag was preserved at room temperature without pressurization in our safety cabinet (control sample), and the other five were pressurized under different conditions. We prepared 5 pressurization conditions in this study: 150, 160, 170, 180, and 190 MPa for 10 min. In brief, each bag was placed in a sample chamber of an isostatic pressurization machine (ECHIGO SEIKA, Co., Ltd., Nagaoka, Japan), and the chamber was filled with tap water. The pressure was increased up to the target pressure of 150, 160, 170, 180, or 190 MPa. The target pressure was then maintained for 10 min, after which the pressure was reduced immediately. The suspensions, including the nonpressurization group (control sample), were then moved to 1.5 mL micro tubes (Scientific Specialties, Inc., Lodi, CA, USA) and centrifuged at 200 g for 5 min. The medium was discarded, and 200 *μ*L of working solution of live/dead staining was added to resuspend the cells. This suspension was incubated for 15 min in the dark and then centrifuged at 200 g for another 5 min. The medium was discarded, and the cells were then suspended again using 200 *μ*L of the 0.6% paraformaldehyde solution and incubated for at least 15 min in the dark. After this, fluorescence micrographs were taken using a fluorescence microscope (BZ-9000; Keyence Corp., Osaka, Japan).

### 2.5. Morphological Observation and a Proliferation Assay of the Cells after Pressurization

Cryopreserved cSCC cells were rapidly thawed, and 1 × 10^6^ cells were seeded on a 10- or 15 cm culture dish and cultured until subconfluence, as mentioned above. Cells were dissociated using the TrypLE™ Express, and cell suspensions of 1 × 10^5^ cells/mL in the respective culture medium were prepared. A total of 5 mL of cell suspension was then packed in a plastic bag, and 6 bags were prepared. One bag was preserved at room temperature without pressurization in our safety cabinet (control sample), and the other five were pressurized under different conditions.

We prepared five pressurization conditions, and each bag was pressurized as mentioned above. A 100 *μ*L aliquot of each cell suspension, including the nonpressurization group, was then seeded into the well of a 24-well cell culture plate (Corning, Inc., Corning, NY, USA) with 1 mL of the respective culture medium. Cells were cultured at 37°C, 95% humidity, and 5% carbon dioxide for 7 days without changing the medium. The cell morphology and attachment were observed at 3 h, 1 day, 3 days, and 7 days after seeding using the inverted microscope (Carl Zeiss Co., Ltd., Oberkochen, Germany).

In addition, the proliferation was evaluated quantitatively using a WST-8 (4-[3-(2-methoxy-4-nitrophenyl)-2-[4-nitrophenyl]-2H-5-tetrazolio]-1,3-benzene disulfonate sodium salt) assay (Cell Counting Kit-8; Dojindo, Kumamoto, Japan). In brief, a 100 *μ*L aliquot of each cell suspension, either after pressurization or without pressurization, was added to each well (*n* = 36, every 4 wells) of a 96-well plate (Corning, Inc.) with 100 *μ*L of the respective culture medium. The plates were then incubated at 37°C in a humidified atmosphere of 5% CO_2_ for 3 h, 1 day, 3 days, or 7 days without changing medium. At each evaluation time point, 10 *μ*L of the WST-8 assay reagent was added to each well and incubated at 37°C for 1 h. Then, the plate was gently shaken, and the absorbance of the medium (*n* = 6 at each point) was determined using a multiplate reader at a wavelength of 450 nm. The absorbance of each medium in the vacant wells (*n* = 6) was also measured, and this absorbance was used as an arbitrary zero point.

### 2.6. Growth of the cSCC Cells after HHP *In Vivo*

A suspension of 10^6^ cells/mL cSCC cells was prepared in the culture medium. The cell suspension was then packed in a plastic bag, and two bags were prepared. One bag was preserved at room temperature without pressurization in our safety cabinet (control group: *n* = 10), and the other was treated with HHP at 200 MPa for 10 min (pressurized group: *n* = 7). Suspensions (200 *μ*L) including 10^6^ cells/mL cells of the pressurized group and nonpressurized group were injected into the intradermal space on the back of 8-week-old male NSG mice using 20 G needles (Terumo Corp., Tokyo, Japan) with 100 mL syringes (Terumo Corp.). During injection, the mice were anesthetized by the inhalation of 2% isoflurane (Wako Pure Chemical Industries, Ltd., Osaka, Japan). The mice were sacrificed by carbon dioxide inhalation, and each specimen was taken at nine weeks after injection. Each formed cSCC tumor (control group) was divided into three pieces: one for the histological assessment and the others for experiments on the growth of the cSCC tumor after HHP *in vivo*.

### 2.7. Growth of the cSCC Tumor after HHP *In Vivo*

The formed cSCC tumors (*n* = 9) were divided into small pieces, and the tumor weight was controlled to approximately 0.5 g. These specimens were preserved in plastic bags with normal saline solution (NSS; Fuso Pharmaceutical, Tokyo, Japan). Half of them were preserved at room temperature without HHP (*n* = 9), and the other half were pressurized at 200 MPa for 10 min (*n* = 9). The tumors were implanted into the subcutis on the backs of 8-week-old male mice. During implantation, the mice were anesthetized by the inhalation of 2% isoflurane. The mice were sacrificed by carbon dioxide inhalation, and specimens were observed, collected, and weighed at five weeks after implantation.

### 2.8. Sampling and the Histological Assessment of cSCC Tumors with HHP

Hematoxylin and eosin (HE) staining, p63 immunohistochemical staining, and Ki-67 immunohistochemical staining of the cSCC tumors were conducted nine weeks after cell injection and five weeks after tumor implantation. Immunohistochemical staining of p63 was performed to detect cSCC cells. The immunohistochemical staining of Ki-67 was performed to detect the cells with excellent proliferation. Regarding the cSCC tumors at nine weeks after injection, the specimens of the control group were collected from the back skin within 5 mm of the injection site, while the specimens of the pressurized group were collected from the tumor region, taking care to capture as little skin as possible. Regarding the cSCC tumors at five weeks after implantation, the specimens were collected from the tumor region, taking care to capture as little skin as possible. The specimens of cSCC tumors five weeks after implantation without HHP were collected and divided into small pieces. These specimens were fixed with 10% formalin-buffered solution (Wako Pure Chemical Industries, Ltd.) and then embedded in paraffin blocks. Sections of 5 *μ*m thickness from the central area of each sample were stained with HE.

For p63 staining, sections were deparaffinized and rehydrated, and then heat-induced antigen retrieval was performed in 10 mM sodium citrate buffer solution (pH 6.0) at 125°C for 30 sec. After cooling to room temperature, the sections were rinsed in distilled water (DW) and 3% hydrogen peroxide for 10 min to block endogenous peroxidase activity. The sections were then rinsed in DW and Tris-buffered saline with Tween-20 and 0.15 M NaCl (TBST), and mouse monoclonal anti p63 antibody (code: 413751; Nichirei Biosciences Inc., Tokyo, Japan) was applied to sections with incubation at room temperature for 30 min. The sections were then rinsed in TBST again, after which Simple Stain MAX-PO(M)® (Nichirei Biosciences Inc.) secondary antibody was applied at room temperature for 30 min. The sections were rinsed in TBST again, exposed to DAB (3-30-diaminobenzidine tetrahydrochloride; Nichirei Biosciences Inc.), and counterstained with hematoxylin. For Ki-67 staining, sections were deparaffinized and rehydrated, and then heat-induced antigen retrieval was performed in EDTA at 98°C for 40 min. After cooling to room temperature, the sections were rinsed in DW and 3% hydrogen peroxide for 10 min to block endogenous peroxidase activity. The sections were rinsed in DW and TBST, and rabbit monoclonal anti-Ki-67-antibody (code: 718071, Nichirei Biosciences Inc.) was applied to sections and incubated at room temperature for 30 min. The sections were then rinsed in TBST again, after which Simple Stain MAX-PO(R)® (Nichirei Biosciences Inc.) secondary antibody was applied at room temperature for 30 min. The sections were rinsed in TBST again, exposed to DAB, and counterstained with hematoxylin. After staining, histologic photographs were taken and analyzed using a NanoZoomer 2.0-HT whole-slide scanner with the NDP.view2 software program (Hamamatsu Photonics, Hamamatsu, Japan) at 40x magnification. The Ki-67-positive cells (control group: *n* = 10, pressurized group: *n* = 7) were identified using the Hybrid Cell Count image analysis program (Keyence Corp.)

### 2.9. Statistical Analyses

Statistical significance was assessed using the Steel test, Mann-Whitney *U* test, and Wilcoxon's signed-rank test. All data are expressed as the mean ± standard deviation. The Microsoft Excel software program (Microsoft Corp., Redmond, WA, USA) with the Statcel software add-on (OMS Publishing, Inc., Tokyo, Japan) was used for all statistical analyses. The Steel test was used to compare cells' proliferation, the Mann-Whitney *U* test was used to compare the Ki-67 index, and Wilcoxon's signed-rank test was used to compare the weight of the two groups. *P* < 0.05 and *P* < 0.01 represent the level of significance. A *P* value < 0.05 was considered statistically significant.

## 3. Results

### 3.1. Live/Dead Cell Staining without Pressurization and after Pressurization

**Figure 1:**
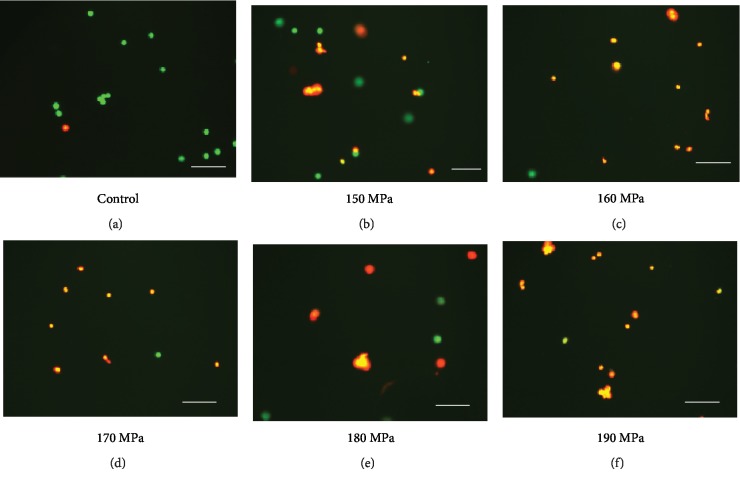
The live/dead staining images of cSCC cells after HHP. The image of the control group (without pressurization) is shown at (a). (b–f) show the images after HHP at 150 MPa, 160 MPa, 170 MPa, 180 MPa, and 190 MPa for 10 min, respectively. Scale bars = 100 *μ*m.

The live/dead staining of cSCC cells showed that most of the cells in the control group were stained by the green fluorescence derived from SYTO 10 green fluorescent nucleic acid (Figure 1(a)). The percentage of cSCC cells stained by the red fluorescence derived from the ethidium homodimer II nucleic acid stain increased with increasing pressure, and the cSCC cells were largely inactivated beyond 190 MPa (Figure 1(f)).

### 3.2. Morphological Observation of cSCC Cells after HHP and the WST-8 Assay

**Figure 2:**
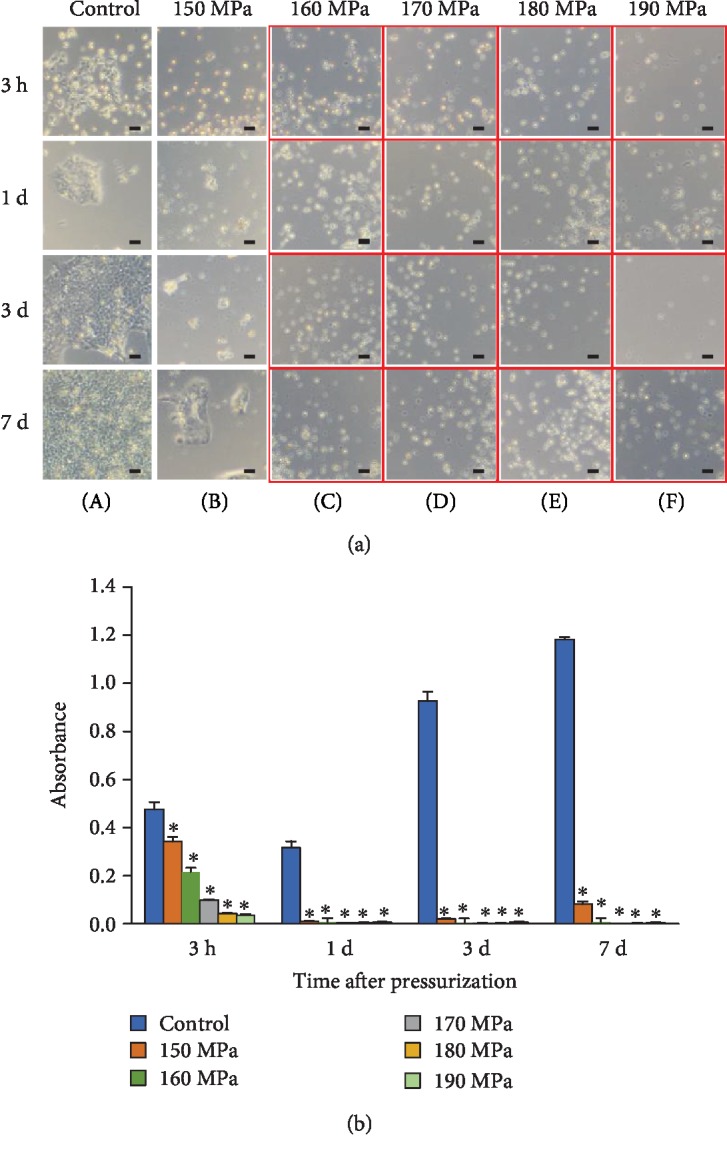
Morphological observation of cSCC cells after HHP and the WST-8 assay. (a) Phase-contrast micrographs of the cSCC cells in the control group and after HHP. Column A shows the micrographs of cSCC cells in the control group at 3 h, 1 day, 3 days, and 7 days after incubation, from top to bottom, respectively. The columns show the micrographs of cells after HHP at 150 MPa,160 MPa, 170 MPa, 180 MPa, and 190 MPa for 10 min (B to F, respectively) at 3 h, 1 day, 3 days, and 7 days after incubation, from top to bottom, respectively. cSCC cells in the red frame are detached and floating. Scale bars = 50 *μ*m. (b) Quantification analyses of cells using the WST-8 assay. The time course of cell viability at 3 h, 1 day, 3 days, and 7 days after incubation is shown in the control group and HHP group for 10 min. ^∗^*P* < 0.05 vs. control.

The micrographs of the cultured cells of the control group and pressurization groups are shown in Figure 2(a). In the control group and pressurization group after HHP at 150 MPa, the cSCC cells were attached to the dish and proliferated continuously for 7 days. In the pressurization group after HHP at ≥160 MPa, however, all cells were floating.

The results of the objective evaluation of cell viability using the WST-8 assay are shown in Figure 2(b). The growth of cSCC cells was disturbed after HHP 150 MPa to some extent and completely inhibited after HHP at ≥160 MPa.

### 3.3. Growth of the cSCC Cells after HHP *In Vitro*

**Figure 3:**
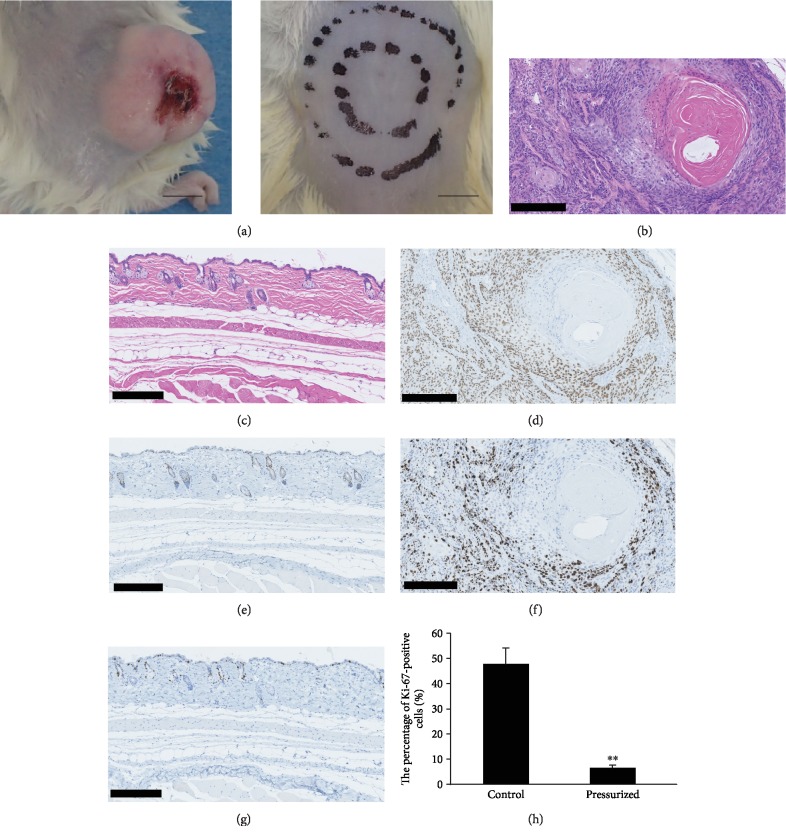
The specimens at nine weeks after injection of inactivation of cSCC cells by HHP. (a) The gross appearance of the back of mice nine weeks after cSCC cell injection without pressurization (control) on the left side and with pressurization (pressurized group) on the right side. Scale bars = 1 cm. (b, c) HE staining, (d, e) p63 immunohistochemical staining, and (f, g) Ki-67 immunohistochemical staining of the specimens injected with cSCC cells without pressurization ((b, d, f) control group) and with pressurization ((c, e, g) pressurized group). Scale bars = 250 *μ*m. (h) The percentage of Ki-67-positive cells in each slide was calculated using an image analysis software program. ^∗∗^*P* < 0.01.

The growth of cSCC tumors nine weeks after injection of cells with pressurization (pressurized group) or without pressurization (control group) is shown in Figure 3. Regarding the macroscopic appearance, cSCC tumors were formed at the injected site in the control group at nine weeks. In contrast, tumors were not observed in any pressurization group (Figure 3(a)). Therefore, the tumor weight of the pressurized groups could not be evaluated (mean control group tumor weight ± standard deviation: 5.98 ± 5.50 g). HE-stained sections of the control group showed large cells with eosinophilic cytoplasm, squamous epithelial cells forming large nests and keratin pearls. Most tumor cells were positive for p63 with an average Ki-67 index (Figures 3(b), 3(d), and 3(f)). In contrast, the HE-stained sections of the pressurized groups showed that the specimens had no tumor regions (Figure 3(c)). The normal skin basal cells were positive for p63 (Figure 3(e)). The percentage of Ki-67-positive cells (Figure 3(h)) was 47.73 ± 6.58% in the control group and 6.41 ± 1.07% in the pressurized group.

### 3.4. Growth of the cSCC Tumors after HHP *In Vivo*

**Figure 4:**
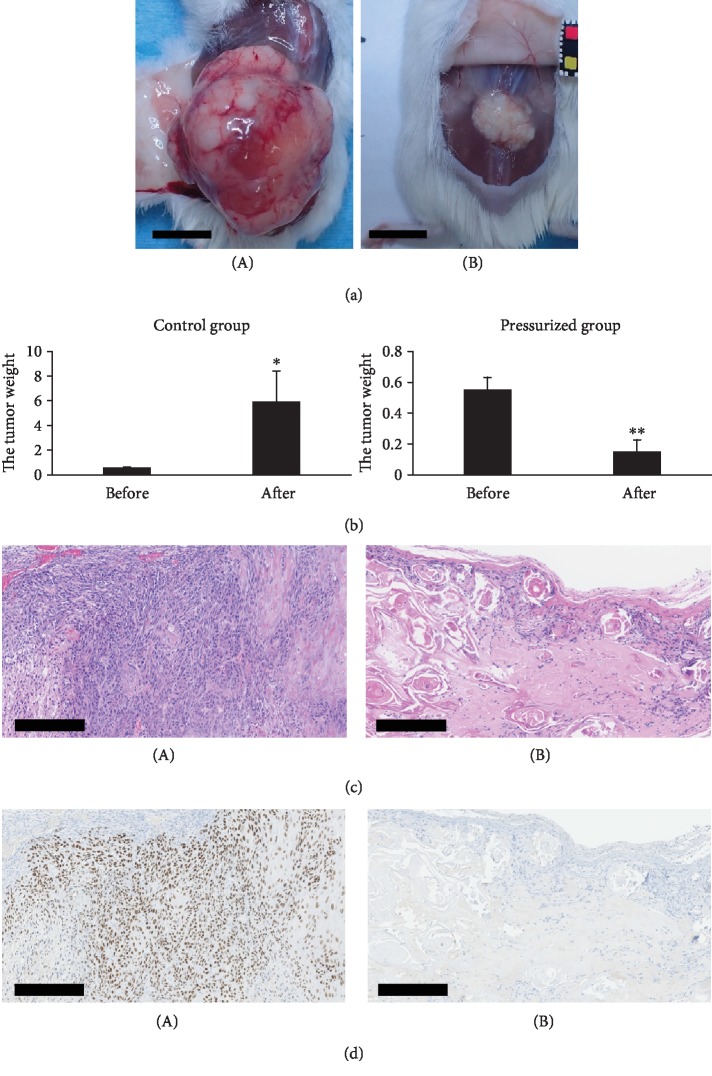
The specimens at five weeks after implantation of inactivation of cSCC cells by HHP. (a) The gross appearances of cSCC tumors five weeks after implantation are shown. The control group is shown in A, and the pressurized group is shown in B. Scale bars = 1 cm. (b) The cSCC tumor was weighed before implantation and five weeks after implantation. ^∗^*P* < 0.05 and ^∗∗^*P* < 0.01. (c) HE staining and (d) p63 immunohistochemical staining of the specimens implanted with cSCC cells without pressurization (control group: A) and with pressurization (pressurized group: B). Scale bars = 250 *μ*m.

One sample in the group without HHP was excluded because of a protruding transplanted tumor on the first day. The gross appearances of the cSCC tumors five weeks after implantation are shown in Figure 4(a). The cSCC tumors in the control group grew rapidly, whereas those in the pressurized groups showed marked regression (Figure 4(a)). The tumor weight at 5 weeks after implantation increased significantly, reaching about 10 times larger than the original size, while that of the pressurized group decreased significantly, reaching approximately one-quarter of the original size (Figure 4(b)).

HE-stained sections of the tumors in the control group showed large cSCC cells with eosinophilic cytoplasm and well-developed keratinization. The HE-stained sections of the pressurized groups showed cells that had degenerated keratin material and foreign body macrophages with no viable tumor cells detected (Figure 4(c)). p63-stained sections of tumors in the control group showed that most cSCC cells were positive for p63, whereas no cSCC cells were clearly detected on p63-stained sections of the pressurized groups (Figure 4(d)).

## 4. Discussion

In this study, we explored the critical pressurization condition necessary to inactivate cSCC cells *in vitro* and *in vivo*. Regarding the inactivation of human malignant cells by HHP, Korn et al. found that pressures from 150 to 250 MPa for 5 min induced programmed cell death in most human tumor cell lines, such as human histiocytic lymphoma, the lymphoblast-like human cell from Burkitt's lymphoma Raji, the human T cell leukemia Jurkat, and the human B cell line from acute lymphoblastic leukemia BALL-1 [12]. Fucikova et al. reported that HHP treatment at more than 150 MPa for 10 min induced rapid apoptosis of cells, including acute lymphoblastic leukemia cell lines, ovarian cancer cells, and prostate cancer cells [13]. In our study, it was showed that cSCC cells were killed by HHP at ≥160 MPa for 10 min, which is compatible with previous reports.

In order to confirm the annihilation of cSCC cells by HHP, we examined the inactivation of cSCC by HHP at 200 MPa for 10 min, as this condition was our standard condition for inactivating cells, skin, and nevus tissue [8–11, 14–16]. As we showed that pressurized cSCC cells did not proliferate after injection into the intradermal space of mice, cSCC cells were completely inactivated after HHP. In the replantation study of cSCC tissue itself, cSCC in the pressurized group did not proliferate again. We observed recurrence up to 27 weeks using some animals and confirmed no recurrence in the pressurized group; however, we were unable to share these data, as the volume of the relapsed cSCC in the control group increased too much after 5 weeks.

The limitation of this study is that we showed the annihilation of only a single cell line of cSCC. However, we have already shown that many kinds of cells, such as human keratinocytes, melanocytes, fibroblasts, adipose tissue-derived stem cells, and a cell line of malignant melanoma cells, were inactivated by HHP under the same conditions [17]. Therefore, we expect HHP treatment to be a useful physical treatment that can inactivate all kinds of cells uniformly under the same conditions.

We are now conducting a clinical trial for GCMN using the HHP technique at 200 MPa for 10 min. In this study, we inactivate the nevus tissue itself using HHP at 200 MPa for 10 min and then graft the pressurized nevus to the original site after resection in order to perform reconstruction using autologous dermis. The purpose of this treatment is to reconstruct skin defects after the removal of GCMN using the removed tissue itself. The present findings suggest the possibility of the novel treatment of cSCC using the cSCC tissue itself after inactivation by HHP. We have already reported that cellular debris in the pressurized tissue biodegraded within several weeks after grafting and survived successfully [8, 10, 11]. In the present study, the debris in the pressurized cSCC tissue also biodegraded after grafting (Figure 4(c)). We therefore expect the cSCC tissue inactivated by HHP to be useful as an autograft for reconstruction, similar to GCMN.

In the present study, we confirmed that HHP was able to annihilate cSCC cells and tissue. Our results suggest that HHP is a simple and effective procedure for treating malignant tumor with a low burden on patients.

## 5. Conclusion

HHP at ≥160 MPa for 10 min was able to inactivate cSCC cells, and that at 200 MPa for 10 min was able to annihilate cSCC tumors completely. HHP technology represents a novel option for cancer treatment.

## Data Availability

All data used to support the findings of this study are included within the article.
